# The Plague of Examinations

**Published:** 1919-07-26

**Authors:** 


### THE MATRONS' AND SISTERS' DEPARTMENT.

### THE PLAGUE OF EXAMINATIONS.

Is there any danger that the appointment of sister-tutors may lead to an extension of the examination system in training-schools under which nurses at present are groaning? As a matter of fact, under a well-co-ordinated curriculum, the introduction of a sister trained to teach and with all her energies set free for this ought to obviate the necessity for holding frequent examinations. Education does not consist in continual preparation for undergoing the feverish ordeal of the examination room, nor is nursing one of those subjects which requires their perpetual stimulus.

The modern training-school, planned on progressive lines, ought not to let itself be dominated by theories which are gradually disappearing from higher education. Like the collegiate establishments they begin to emulate, training-schools for nurses possess one immense advantage: the students are all adults. They are all responsible persons who need help, it is true, in prosecuting their studies, but not compulsion. The difference in age which separates one probationer from another may be ten or twelve years, but it is far less in reality than the gulf which separates girls of thirteen and seventeen. The lecturer and teacher may rely on the active co-operation of the pupil's will in the process of imbibing knowledge. Hence the need for continually testing the progress of the whole body of students disappears. Backward pupils may need the discipline of test papers pretty often. Average students do better when they are permitted to master their subjects without the cramping effect of considering for ever whether the matter in hand is likely to count in the eyes of the examiner. It becomes possible to encourage individual tastes and talents, and to let clever students follow up subjects which interest them when once the nightmare worries which neutralise the effects of good work are banished.

There are certain subjects necessary for nurses to master which demand memory tests. The nomenclature of anatomy, of physiology, and pharmacy, is among these. But nurses master names by using them in a far more satisfactory manner than by learning them by heart. Under the latter process, which can be achieved by cram, all is forgotten when the examination is over. The former process leads to understanding, and knowledge so acquired becomes a permanent possession. This distinction has to be kept in mind throughout the entire training of the nurse. That part of the curriculum which can be "worked up" for examination purposes needs the most careful discrimination from the rest, and ought to be the subject of such frequent reiteration from the outset that it becomes a familiar ground plan in traversing which the student is sure because fearless. Continuous *viva voce* questioning is the only road through which this can be attained. And the value of the pupils in relation to each other must not be lost sight of in this connection. Many a nervous muddle-headed probationer first finds confidence in her own powers through having to teach a newcomer something she has lately learned herself, and the principle of learning by teaching is far too good to be confined merely to the practical part of the training.

Unquestionably the sister-tutor ought not to be overburdened with incessantly correcting paper work, nor harried by the ogre of recurring examinations. She gives her own lectures in accordance with the syllabus. She attends and expounds lectures given by the staff. She directs the individual studies of probationers, and illustrates by her own methods the way in which they may test and stimulate each other. But the responsibility for learning must still rest on the pupils. A nurse needs to have her knowledge at her finger tips or it is of no use to her. When the moment comes for using it she will not be provided with a silent room in which to think out her ideas and commit them to pen and ink; she will be confronted with the call to remember what she is trying to master in her student days on some occasion, perhaps, when a life may be at stake, or, at the least, her own reputation for efficiency. To make her education centre round examinations is to set both her and her teacher in bondage. Her theoretical knowledge must become part of herself, must be tested in a hundred practical directions, and once again be mastered under an abstract presentment before it becomes of real use to her. In her final examination she should find herself in a region so familiar as to banish all anxiety.

The training of nurses is in the melting-pot. We cannot tell yet what will emerge under the new conditions which are arising. What is chiefly needed to ensure its development on sound lines is liberty for the teacher and liberty within certain well-defined lines for the student. Probationers are unequal in mind and previous attainments. Let us guard against maiming their intellects in a vain attempt at uniformity.

